# Evaluation of the Deletion of the African Swine Fever Virus Gene *O174L* from the Genome of the Georgia Isolate

**DOI:** 10.3390/v15102134

**Published:** 2023-10-23

**Authors:** Elizabeth Ramirez-Medina, Lauro Velazquez-Salinas, Ayushi Rai, Nallely Espinoza, Alyssa Valladares, Ediane Silva, Leeanna Burton, Edward Spinard, Amanda Meyers, Guillermo Risatti, Sten Calvelage, Sandra Blome, Douglas P. Gladue, Manuel V. Borca

**Affiliations:** 1 Plum Island Animal Disease Center, ARS, USDA, Greenport, NY 11944, USA; elizabeth.ramirez@usda.gov (E.R.-M.); lauro.velazquez@usda.gov (L.V.-S.); ayushi.rai@usda.gov (A.R.); nallely.espinoza@usda.gov (N.E.); alyssa.valladares@usda.gov (A.V.); ediane.silva@usda.gov (E.S.); leeanna.burton@usda.gov (L.B.); edward.spinard@usda.gov (E.S.); amanda.meyers@usda.gov (A.M.); 2 Oak Ridge Institute for Science and Education (ORISE), Oak Ridge, TN 37830, USA; 3 Department of Pathobiology and Veterinary Science, University of Connecticut, Storrs, CT 06269, USA; guillermo.risatti@uconn.edu; 4 Friedrich-Loeffler-Institut, Federal Research Institute for Animal Health, Südufer 10, 17493 Greifswald-Insel Riems, Germany; sten.calvelage@fli.de (S.C.); sandra.blome@fli.de (S.B.)

**Keywords:** ASFV, ASF, ASFV, O174L, African swine fever virus, virus virulence, viral replication

## Abstract

African swine fever virus (ASFV) is a structurally complex, double-stranded DNA virus, which causes African swine fever (ASF), a contagious disease affecting swine. ASF is currently affecting pork production in a large geographical region, including Eurasia and the Caribbean. ASFV has a large genome, which harbors more than 160 genes, but most of these genes’ functions have not been experimentally characterized. One of these genes is the *O174L* gene which has been experimentally shown to function as a small DNA polymerase. Here, we demonstrate that the deletion of the *O174L* gene from the genome of the virulent strain ASFV Georgia2010 (ASFV-G) does not significantly affect virus replication in vitro or in vivo. A recombinant virus, having deleted the *O174L* gene, ASFV-G-∆O174L, was developed to study the effect of the *O174L* protein in replication in swine macrophages cultures in vitro and disease production when inoculated in pigs. The results demonstrated that ASFV-G-∆O174L has similar replication kinetics to parental ASFV-G in swine macrophage cultures. In addition, animals intramuscularly inoculated with 10^2^ HAD_50_ of ASFV-G-∆O174L presented a clinical form of the disease that is indistinguishable from that induced by the parental virulent strain ASFV-G. All animals developed a lethal disease, being euthanized around day 7 post-infection. Therefore, although O174L is a well-characterized DNA polymerase, its function is apparently not critical for the process of virus replication, both in vitro and in vivo, or for disease production in domestic pigs.

## 1. Introduction

African swine fever, a frequently lethal disease that affects wild and domestic pigs, is currently present in large parts of Europe, Asia, and more recently the Caribbean area [1]. No commercial vaccines are available outside of Vietnam; therefore, the control of the disease in most countries is based on culling all infected animals and restricting the mobility of susceptible animals.

The causative agent of the disease, African swine fever virus (ASFV), is a large and structurally complex virus, harboring a 180–190 kilobase pairs genome composed of a double-stranded DNA that encodes more than 160 genes [2,3]. Although several of these genes have been recently characterized, the role of many of them in critical functions such as virus replication or disease production remains unknown. The study of gene function using recombinant ASFV having deleted individual genes has been a critical tool to identify the role of particular genes in different aspects of the virus cycle. The deletion of some of the virus genes produces small or undetectable phenotypic changes in virus replication or virulence during the infection in pigs (i.e., A859L [4], KP177R [5], MGF110-1L [6], X69R [7], MGF360-1L [8], and CD2 [9]), while in other cases, the deletion induces dramatic differences in the phenotype of the parental virus: I177L [10,11], 9GL [12], I226R [13], A137 [14], I267L [15], MGF505-7R [16], and MGF110-9L [17]. In fact, the discovery of virus genes implicated in the process of ASFV virulence in pigs was fundamental for the production of live attenuated vaccine strains that are efficacious in inducing protection against the infection with the corresponding virulent parental field isolate [10,11,12,13,14,15,16,17,18]. 

In this report, we characterize the effect of deleting the *O174L* gene from the genome of the highly virulent ASFV isolate Georgia 2010 (ASFV-G) in the process of virus replication and virus virulence in experimentally infected domestic pigs. The *O174L* gene has long been recognized to encode a small DNA polymerase, pol X [19,20]. This protein has been shown to repair single-nucleotide gapped DNA substrates [19]. A recombinant virus lacking the *O174L* gene in the genetic background of the field-isolated BA71 adapted to grow in Vero cells (BA71V) has been shown to have a significantly decreased replication in swine macrophages when seeded at a low MOI [20]. The potential importance of O174L has led to studies on potential targeting for biotherapeutics to inhibit virus replication [21].

We demonstrate here that a recombinant virus harboring a deletion of the *O174L* gene from the genome of virulent ASFV-G isolate (ASFV-G-∆O174L), when compared with the virulent parental, has a similar ability to replicate in swine macrophages cultures; additionally, when experimentally inoculated in swine, its virulence appears similar to that of the ASFV-G strain.

## 2. Materials and Methods

### 2.1. Viruses and Cells

The parental ASFV strain was isolated in the Republic of Georgia in 2010 (ASFV-G) and was kindly provided by Dr. Nino Vepkhvadze from the Laboratory of the Ministry of Agriculture, Tbilisi, Republic of Georgia. Primary macrophage cell cultures were produced as described previously [22], and in all the experiments performed in this study, they were seeded at a concentration of 5 × 10^6^ cells/mL. Virus titrations, virus stock production, as well as transfection/infections were performed using swine macrophage cultures as described [22]. Comparative growth kinetics between the ASFV-G-∆O174L and ASFV-G strain were set using an MOI of 0.01 HAD_50_ as previously described [22] with sample points obtained at 2, 24, 48, 72, and 96 h post infection and titrated using swine macrophage cell cultures in 96-well plates. Virus-infected cells were detected through hemadsorption (HA) and the virus titers calculated using the Reed and Muench method [23].

### 2.2. Detection of O174L Transcription

The transcriptional kinetics of the *O174L* gene were assessed using real-time PCR (qPCR) in cultures of primary swine macrophage, which were infected at an MOI = 10 with ASFV-G. Two well-studied ASFV genes, the early CP204L (p30) and the late B646L (p72) genes, were used as a control for transcription kinetics. After infection, RNA was extracted using an RNeasy Kit (QIAGEN, Hilden, Germany) at 0, 1, 2, 3, 4, 5, 6, 7, 8, 9, and 24 h post infection. The extracted materials were then treated with 2 units of DNase I (Bio Labs, Ipswich, MA, USA) and purified with the Monarch^®^ RNA Cleanup Kit (New England BioLabs, Inc., Ipswich, MA, USA). After DNase treatment, the CT value was over 36, indicating that the DNAase treatment worked. Then, 1 µg of RNA was used to produce the cDNA using qScript cDNA SuperMix (Quanta bio, Beverly, MA, USA), which was also used for the qPCR. The primers and probe for the detection of the *O174L* gene were designed using the ASFV Georgia 2007/1 strain (GenBank Assession # NC_044959.2). Primers forward: 5′- CTGCCCAA CATTCGCATAAAG-3′, reverse: 5′- ACACTTTCGTTCTCCGCAGACTTTTAC A-3′, and probe: 5′-FAM//MGBNFQ-3′. Primers and probes for the detection of p72, p30, and the β-actin gene were previously described [24]. All qPCRs reactions were performed using the TaqMan Universal PCR Master Mix (Applied Biosystems) under the following amplification conditions: one step at 55 °C for 2 min, followed by one denaturation step at 95 °C for 10 min, 40 cycles of denaturation at 95 °C for 15 s, and annealing/extension at 65 °C for 1 min.

### 2.3. Construction of the ASFV O174L Deletion Mutant

An ASFV-G virus harboring the deletion of the *O174L* gene(ASFV-G-∆O174L) was developed by homologous recombination between the ASFV-G genome and a recombination transfer vector following procedures previously described [18]. The recombinant transfer vector (p72mCherryΔO174L) harbors the left and the right flanking genomic regions of the *O174L* gene: the left region extends between genomic positions 128,220–129,220 while the right region is located between genomic positions 129,746–130,746 and also harbors the reporter gene cassette containing the mCherry fluorescent protein (mCherry) gene under the control of the ASFV p72 gene promoter [18]. The recombinant transfer vector was commercially synthesized (Epoch Life Sciences, Sugar Land, TX, USA). This design produces a 525-nucleotide deletion between nucleotide positions 129,221 and 129,745, completely deleting the *O174L* gene. The recombinant ASFV-G-∆O174L was further purified by limiting dilution based on the detection of the activity of the mCherry. The ASFV-G-∆O174L stock was full-length sequenced using next-generation sequencing (NGS).

### 2.4. Next-Generation Sequencing of ASFV

Virus DNA from the infected macrophage cultures that showed 90–100% CPE was obtained using the Nuclear Extract Kit (Active Motif, Carlsbad, CA, USA). After separation from the nucleus, the cytoplasmic fraction was used to obtain the viral DNA by following the manufacturer’s protocol. Briefly: virus-infected cells were harvested and treated with the hypotonic buffer on ice for 15 min. Then, the fraction containing the nucleus was separated using centrifugation, the cytoplasmic fraction was collected, and the DNA was extracted by adding 10% 3 M NaOAc by volume to the sample (Sigma-Aldrich, St. Louis, MO, USA) and an equal volume of phenol:chloroform:isoamyl alcohol (25:24:1) with a pH of 6.5–6.9 (Sigma-Aldrich). These were then centrifuged at maximum speed in a tabletop centrifuge. Then, the aqueous phase was precipitated using 2 volumes of 100% ethanol, washed with the same volume of 70% ethanol, and dried. The obtained pellet of DNA was then resuspended in sterile water. The DNA library was then used for NGS sequencing using Nextera XT kit in the NextSeq sequencer (Illumnia, San Diego, CA, USA), strictly following the manufacturer’s protocol. Sequence analysis was performed using CLC Genomics Workbench software version 22 (CLCBio, Waltham, MA, USA).

### 2.5. Evaluation of ASFV-G-ΔO174L Virulence in Domestic Pigs

ASFV-G-∆O174L virulence was assessed in domestic pigs. Pigs were procured from a commercial vendor of swine specifically for experimental use. The animals used were female Yorkshire pigs vaccinated at the vendor facility for common pathogens of swine; they weighted 35–40 kg at delivery. After delivery, the pigs were allowed 2 weeks of acclimation in the facility prior to the start of experiments. Groups of pigs (*n* = 5) were intramuscularly (IM) inoculated with 10^2^ HAD_50_ of either ASFV-G-∆O174L or the virulent ASFV-G strain. Appearance of clinical signs (such as depression, anorexia, staggering gait, purple skin discoloration, diarrhea, and cough) as well as changes in body temperature were recorded daily. Blood samples were scheduled to be obtained at days 0, 4, 7, 11, 14, 21, and 28 post-inoculation (pi). All animal experiments were performed under biosafety level 3 conditions in the animal facilities at Plum Island Animal Disease Center, strictly following a protocol approved by the Institutional Animal Care and Use Committee (225.06-19-R_090716, approved on 9 June 2019).

### 2.6. Read Mapping and Variant Detection

All steps were performed using CLC Genomics Workbench v23 (QIAGEN, Aarhus, Denmark). Illumina reads were trimmed for quality (limit = 0.05), ambiguous base pairs (max = 2), adapters, and size (min = 100 and max = 15) and were mapped against ASFV Georgia 2007/1 (Genbank accession: FR682468.2) using the “Map Reads to Reference” tool with the following parameters: Masking Mode = No Masking, match score = 1, mismatch score = 2, gap cost = linear, insertion and deletion cost = 3, length fraction = 0.5, similarity fraction = 0.8, global alignment = off, auto-detect paired distances = on, and non-specific match handling = random. Basic Variant detection was then performed on the read mappings using the following parameters: Ploidy = 2, ignore positions with coverage over 2,000,000, ignore broke pairs = off, ignore non-specific matches = off, minimum coverage = 1, minimum count = 1, minimum frequency = 50%, and filters for quality, direction/position, and technology specifics = off. Single-nucleotide polymorphisms (SNPs) that appeared in over 50% of reads were considered to be of high confidence in this study; there were no SNPs observed.

## 3. Results and Discussion

### 3.1. Evolution of O174L Gene in Nature

**Figure 1:**
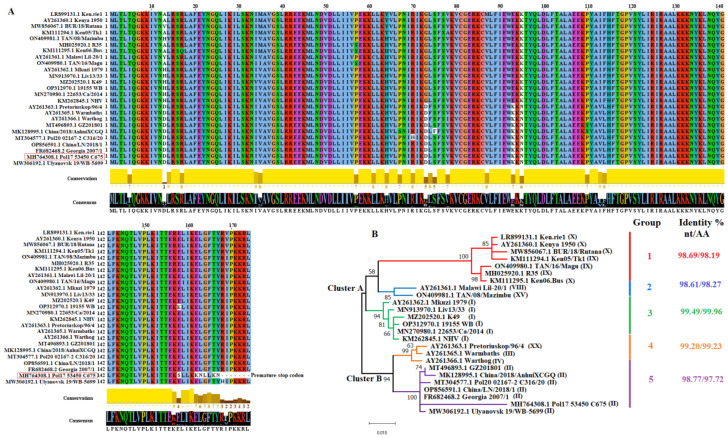
Diversity of the ASFV O174L protein in nature. (**A**) Amino acid alignment representing the diversity of O174L protein of ASFV in the field. Residues in white spots represent changes between amino acids with different charges. Conservation plot scores reflect the nature of the change in specific sites. High scores are associated with changes with similar biological properties. Alignment was produced using the software Jalview version 2.11.1.4. (**B**) Phylogenetic analysis conducted using the full-length sequence of O174L. Numbers in parenthesis represent the genotype classification of different isolates based on P72 gene. Nucleotide (nt) and Amino acid (AA) values of identity were predicted within diverse phylogenetic groups. Analysis was conducted using the MEGA software version 10.2.5 [28].

To gain more insights into the evolutionary dynamic of the *O174L* gene in nature, we conducted a comprehensive evolutionary analysis as previously described for SARS-CoV-2 [25]. First, to obtain a representation of the genetic diversity of the *O174L* gene in nature, a blast analysis was conducted using a version of this gene from the ASFV isolate Georgia 2007/1 as query (GenBank Access FR682468.2). As a result, a total of 25 viral sequences were retrieved from the GenBank database. These sequences included genotypes I (*n* = 6), II (*n* = 7), III (*n* = 1), IV (*n* = 1), VIII (*n* = 1), IX (*n* = 3), X (*n* = 4), XV(*n* = 1), and XX (*n* = 1) (Figure 1). Based on a pairwise analysis, we predicted a nucleotide and amino acid identity of about 89.98–99.80% (~95.92%) and 88.62–99.40% (~95.80%), respectively, showing the high levels of conservation of this gene in nature. However, recently, in Poland, ASFV strains were found carrying a unique phenotype of this protein that included the presence of a 14-nucleotide insertion [26], introducing a premature stop codon and changing the amino acid composition close to c-terminus of this protein (Figure 1A). Interestingly, the presence of this insertion has been useful to track the origin of ASFV strains circulating in Poland [27]. Phylogenetic reconstruction analysis using the maximum likelihood method and the Tamura 3-parameter as a substitution model (AIC score = 2792.043) [28] revealed the existence of two main clusters (A and B) from which diverge at least five distinct phylogenetic groups (Figure 1B). In this context, despite the relatively high conservation at the nucleotide and amino acid levels within groups (with levels of amino acid identity of about 93% between strains comprising groups 1 and 5), it may be possible that there exists potential functional differences between *O174L* gene phenotypes in nature. The latter may be supported based on the amino acid substitutions predicted at residues 13, 73, 93, and 95, where conservation plot scores of 1, 5, 7, and 6, respectively, reflect changes in the biological properties (charges) between replacements.

Functionally, the *O174L* gene of ASFV has been described as a reparative DNA polymerase (Pol X), with a potential role in the preservation of viral genetic information during replication in infected cells [20]. In this sense, critical residues previously determined with a potential function linked to catalysis and fidelity (Ser-39, Arg-42, Asp-49, Asp-51, Glu-83, Lys-85 and Asp-100) [29] were found totally conserved among multiple isolates.

**Figure 2:**
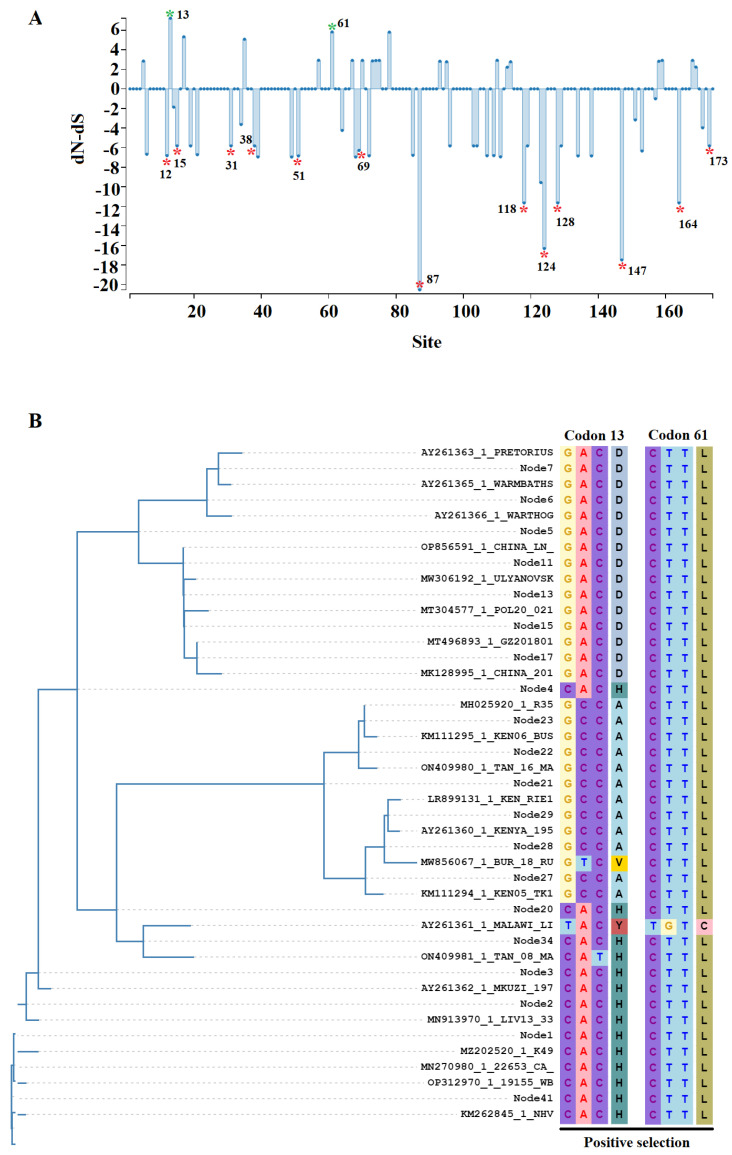
Evolutionary dynamics of *O174L* gene in nature. (**A**) The graphic represents the dN (rate of evolution at non-synonymous sites) dS (rate of evolution at synonymous sites) ratio (dN − dS) at specific codon sites in the *O174L* gene of ASFV. Green asterisks represent codons detected under positive selection by the algorithms MEME and FUBAR (cutoff values of *p* = 0.1, and posterior probability = 0.9, respectively). Red asterisks represent codons detected by FEL (cutoff value of *p* = 0.1) under negative selection (the amino acids encoded by these codons can be visualized in Figure 1A) (**B**) Ancestral reconstruction conducted by the algorithm Single Likelihood Ancestor Counting (SALC) [29], representing the codons predicted under positive selection using FUBAR (Codon 13) and MEME (Codon 61) analyses.

The evaluation of the *O174L* gene sequences using the algorithm fixed effects likelihood (FEL) [30] indicated that a purifying selection is the main force driving the evolution of this gene (dN/dS = 0.287). In this sense, the FEL determined a total of 13 codon sites under a negative selection distributed along the gene (Figure 2A), indicating the potential relevance of these sites in the functionality of the O174L protein. Interestingly, one of these sites included the codon encoding the catalytic Asp-51, confirming the relevance of this site during the evolution of ASFV in nature. Considering the collection date of the isolation of K49, 1949, it is possible to infer that these sites have remained conserved for more than 70 years. Furthermore, the identification of these sites may represent a framework for future research intended to identify critical sites in this protein.

Two different codon sites were predicted under a positive selection (Figure 2B), indicating that these codons may provide potential adaptative advantages to ASFV. The first codon (codon 13) was detected using the algorithm Fast Unconstrained Bayesian AppRoximation (FUBAR) [31]. The selection of this codon among multiple internal nodes and different phylogenetic groups represents evidence of pervasive positive selection (Figure 2B). Conversely, evidence of an episodic positive selection impacting a single ASFV isolate (Malawi Lil-20/1) was found in codon 61 using the algorithms mixed effects model of evolution (MEME) [32] (Figure 2B), showing the different modes of positive selection impacting the evolution of the *O174L* gene.

No evidence of potential breakpoints was predicted during the evaluation through the genetic algorithm for recombination detection (GARD) [33], indicating that recombination is not playing a significant role in the evolution of the *O174L* gene in nature.

### 3.2. Detection of O174L Transcription

**Figure 3:**
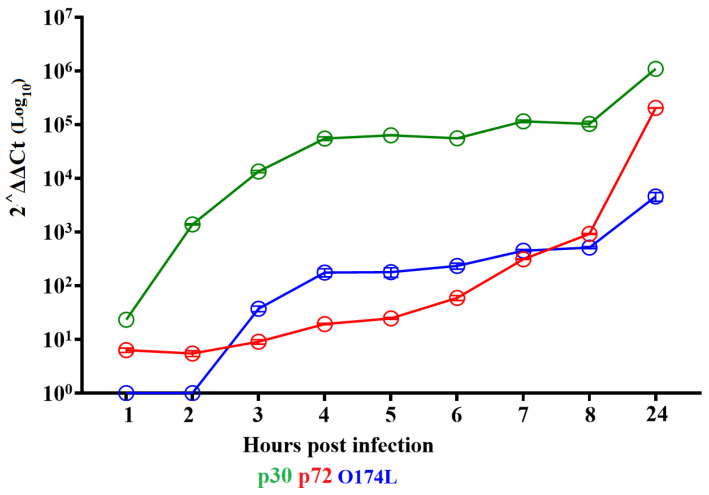
Expression profile of the *O174L* gene of ASFV during in vitro infection of porcine macrophages. Reverse transcription followed by qPCR was used to evaluate the expression profile of the *O174L* gene during in vitro infection at different time points up to 24 h. As a reference for this analysis, we used qPCRs to specifically detect the expression of genes encoding ASFV proteins p30 (early expression) and p72 (late expression). Additionally, the actin gene was used as a control to evaluate the quality and levels of RNA during the infection at different time points. Values are represented as Log 10 of relative quantities of mRNA accumulation, estimated by 2^ΔΔCT^. Circles and bars represent the average and standard deviation between biological replicates from each gene at specific time points. B-actin was used to normalize the expression of ASFV genes at different time points. Samples were tested to be DNA negative after DNase treatment.

To assess the time at which the transcription of the *O174L* gene occurs during the cycle of replication of ASFV, an experiment was performed in primary swine macrophages cultures where samples were taken at different times post infection (pi). Macrophages were infected at an MOI of 10 with ASFV strain Georgia (ASFV-G), and samples were sequentially taken at 1, 2, 3, 4, 5, 6, 7, 8, 9, and 24 h pi (hpi). The presence of *O174L* RNA was detected with the RT-PCR described in the Materials and Methods. The transcription of the *O174L* gene was first detected after 4 hpi and increased progressively until 24 hpi (Figure 3). The pattern of expression of the two well-characterized ASFV genes, the early gene encoding for p30 (CP204L), and the late gene encoding for p72 (B646L), were included as a reference for early and late transcriptions, respectively. The results showed that the *O174L* gene is expressed as a late gene overlapping the kinetics of the late B646L gene.

### 3.3. Development of the ASFV-G-ΔO174L Deletion Mutant

**Figure 4:**
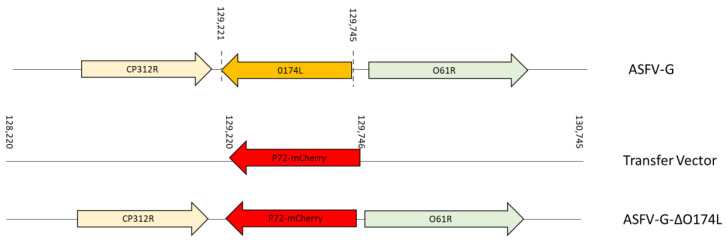
Schematic of the development of ASFV-G-∆O174L. The transfer vector contains the p72 promoter and an mCherry cassette. The gene positions are indicated. The homologous arms were designed to have flanking ends to both sides of the deletion/insertion cassette. The nucleotide positions of the area that was deleted in the ASFV-G genome are indicated by the dashed lines. The resulting ASFV-G-∆O174L virus with the cassette inserted is shown on the bottom.

The conservation of the *O174L* gene and its tested enzymatic function as X-Pol [20,21] would support the hypothesis that O174L can play a critical role in several virus processess such as virus replication and disease production in domestic pigs. To evaluate this hypothesis, a recombinant virus using the ASFV-G strain that harbors a deletion of the *O174L* gene was created through genetic manipulation (ASFV-G-∆O174L). The *O174L* gene was deleted by replacing the complete amino acid residues encoded by the *O174L* gene with the p72mCherry cassette through homologous recombination. An area covering 524 bp between nucleotide positions 129,221 and 129,745 was eliminated from the genome of ASFV-G, completely deleting the *O174L* gene. This deletion was then substituted with a 1226-bp cassette containing the p72mCherry construct (see Materials and Methods) (Figure 4). The recombinant ASFV-G-∆O174L stock was purified through successive limiting dilution steps in primary swine macrophage cell cultures.

The accuracy of the genetic modifications introduced into the ASFV-G-∆O174L genome was evaluated through full genome sequencing obtained via NGS using an Illumina NextSeq^®^ 500, with an average depth of 848 reads. A comparison between ASFV-G-∆O174L and ASFV-G genomes showed a deletion of 525 nucleotides and an insertion of 1226 nucleotides corresponding to the p72-mCherry cassette sequence. No additional unwanted changes were created during the process of the production and purification of ASFV-G-∆O174L. Also, the NGS data showed the absence of ASFV-G genome as indicating the purity of the ASFV-G-∆O174L stock.

### 3.4. Assessment of Replication of ASFV-G-∆O174L in Swine Macrophages Cultures

**Figure 5:**
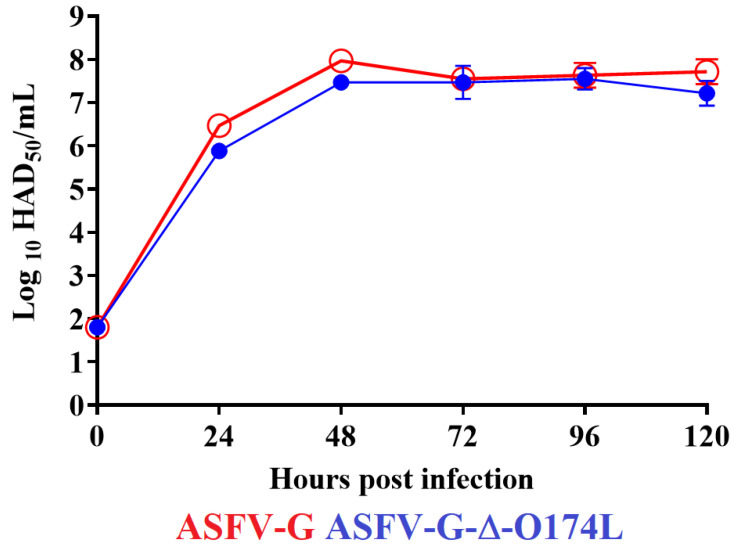
In vitro growth kinetics in primary swine macrophage cell cultures for ASFV-G-∆O174L and parental ASFV-G (MOI = 0.01). Samples were taken from two independent experiments at the indicated time points and titrated. Data represent means and standard deviations of three replicas. Sensitivity using this methodology for detecting virus was ≥log10 1.8 HAD_50_/mL.

To evaluate the potential importance of the *O174L* gene in the process of ASFV replication, the ability of the recombinant ASFV-G-∆O174L to replicate in cultures of primary swine macrophage was compared to that of the parental ASFV-G by plotting a multistep growth curve. Macrophages were infected at an MOI of 0.01 with either ASFV-G-∆O174L or ASFV-G. Virus production was evaluated at 2, 24, 48, 72, and 96 h post infection. The results demonstrated that ASFV-G-∆O174L showed almost indistinguishable kinetics of replication to the parental ASFV-G. No statistical differences were found in any of the time points tested (Figure 5). Therefore, the deletion of the *O174L* gene from the genome of the highly virulent ASFV-G does not affect its replication in primary swine macrophage cultures.

### 3.5. Assessment of ASFV-G-∆O174L Virulence in Swine

**Figure 6:**
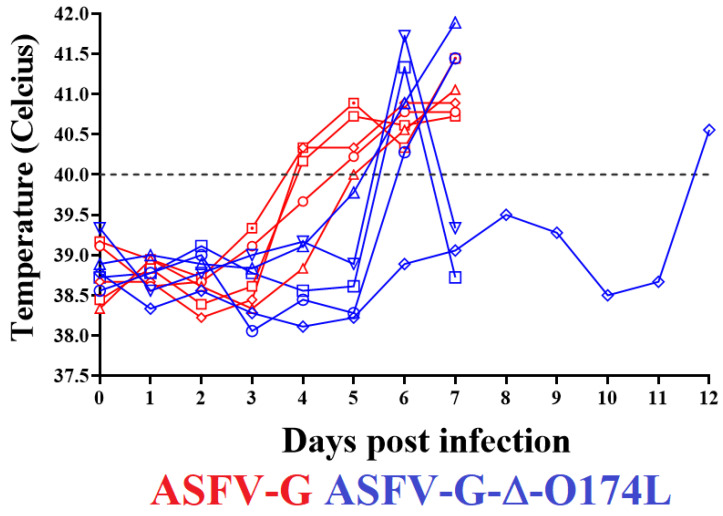
Evolution of body temperature in animals (5 animals/group) IM inoculated with 10^2^ HAD_50_ of either ASFV-G-∆O174L or parental ASFV-G.

**Figure 7:**
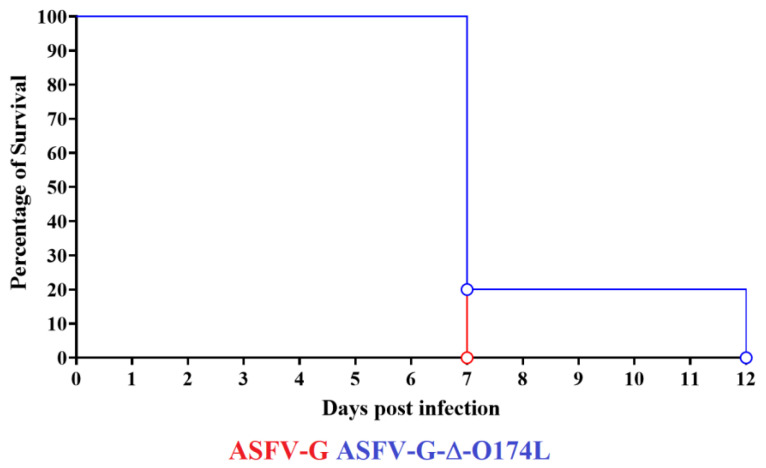
Evolution of mortality in animals (5 animals/group) IM infected with 10^2^ HAD_50_ of either ASFV-G-∆O174L or parental ASFV-G.

To evaluate the effect of the deletion of the *O174L* gene in the virulence of parental ASFV-G in swine, the ASFV-G-∆O174L was inoculated IM at a dose of 10^2^ HAD_50,_ in a group (*n* = 5) of 35–40 kg domestic pigs. The appearance of clinical signs associated with ASF was monitored daily for 28 days. A control group of animals, with similar characteristics, was inoculated IM with 10^2^ HAD_50_ of the parental virulent ASFV-G. All animals inoculated with the virulent ASFV-G showed a sharp increase (over 40 °C) in body temperature by day 4–5 pi, developing a full lethal form of clinical disease (anorexia, depression, diarrhea, staggering gait, and purple skin discoloration) and terminating in all inoculated animals being euthanized by day 6–7 pi due to the severity of the clinical signs (Figure 6 and Figure 7).

Four of the animals inoculated with ASFV-G-∆O174L showed a sharp peak in body temperature (over 40 °C) on day 6 pi with quick worsening of the clinical disease. By day 7 pi, all four animals were euthanized due to the severity of the clinical disease. The fifth remaining animal remained clinically normal until day 10 pi, developing a sharp increase in body temperature accompanied by a sudden presentation of disease on day 11 pi and euthanasia by day 12 pi (Figure 6 and Figure 7).

The genome of the infectious virus isolated at the time of the euthanasia of the animals inoculated with ASFV-G-∆O174L was assessed to ensure that the virulent phenotype observed in those animals was due to the recombinant virus and was not the result of a potential presence of the virulent parental virus contaminating the ASFV-G-∆O174L stock. The results demonstrated that the genome of viruses isolated from all five animals in this group corresponded to the recombinant ASFV-G-∆O174L.

**Figure 8:**
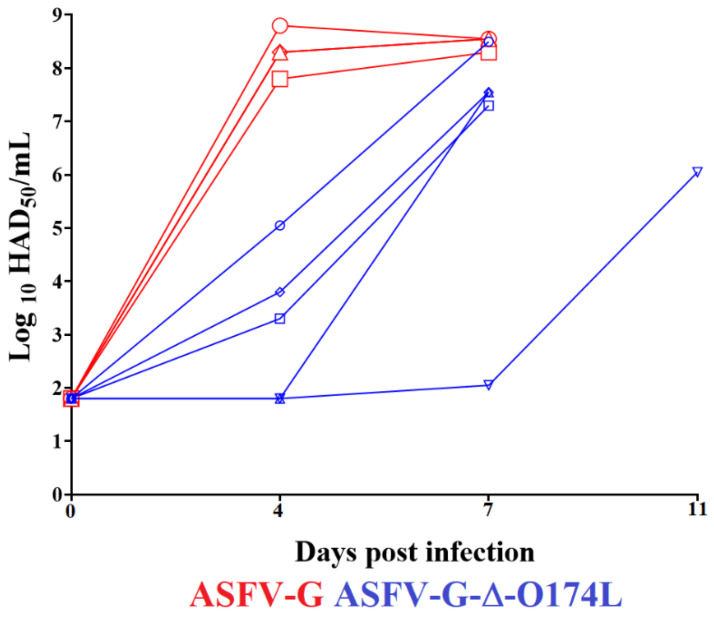
Viremia titers detected in pigs IM inoculated with 10^2^ HAD_50_ of either ASFV-G-∆O174L or ASFV-G. Each symbol represents the data of individual animals in each of the groups. Sensitivity of virus detection: ≥log10 1.8 TCID_50_/mL.

The replication of recombinant ASFV-G-∆O174L in the experimental infected pigs was assessed by quantifying viremia titers after inoculation and comparing them to those detected in animals inoculated with parental ASFV-G. The animals inoculated with ASFV-G exhibited high virus titers (ranging from 10^7.55^–10^8.55^ HAD_50_/mL) by day 4 pi, remaining high until day 7 pi, when all animals were euthanized (Figure 8). Titers of viremia at 4 days pi in animals infected with ASFV-G-∆O174L showed undetectable levels (with a sensitivity of detection of 10^1.8^ HAD_50_/mL) in two of them and titers in the range of 10^3^–10^5.55^ HAD_50_/mL in the other three remaining animals. The viremia values in these three animals sharply increased to titers in the range of 10^7^–10^8.55^ HAD_50_/mL by day 7 pi, when they were euthanized due to the severity of the disease. In addition, one of the two animals showing undetectable viremias at day 4 pi also drastically increased by day 7 (to 10^7.55^ HAD_50_/mL) and was euthanized. The remaining animal showed a slight increase in viremia values, detectable by day 7 pi and increasing progressively until titers of 10^6^ HAD_50_/mL by day 11 pi, when it was euthanized.

The results reported here demonstrate that the deletion of the *O174L* gene in the context of the ASFV-G isolate does not affect virus replication in the natural target cell, swine macrophages. Although viremia values are clearly lower in the initial phase of the infection in animals inoculated with the recombinant virus, those differences are minimized by the time of euthanasia, indicating that the deletion of the *O174L* gene does not significantly alter the production of a lethal form of the clinical disease. Only one out of the five inoculated animals presented a protracted form of the disease with a lethal presentation. This is not a surprising result since the attenuation of the virulence of highly virulent ASFV-G isolate by deleting a single gene is not a common event. Only seven individual gene deletions have been reported to induce full attenuation of ASFV-G (or its derivative isolates) virulence: I177L [10], I226R [13], A137 [14], I267L [15], MGF505-7R [16], MGF110-9L [17], and 9GL [18].

It has been shown that a recombinant virus lacking the *O174L* gene in the genetic background of the field-isolated BA71 adapted to grow in the Vero cell line (BA71V) has a significantly decreased replication in swine macrophages when seeded at a low MOI [20]. The report shows that while no differences in replication kinetics occur between the parental BA71V virus and the recombinant virus lacking the *O174L* gene in Vero cell cultures, the recombinant virus presents a clearly decreased ability to replicate in swine macrophages when compared with the parental virus. This result was not confirmed by our experiment, where we showed that ASFV-G and ASFV-G-∆O174L, seeded at an MOI of 0.01, present undistinguishable growth kinetics.

As occurred in other cases [34,35] where the effect of gene deletion was evaluated using ASF viruses adapted to grow in cell lines, these divergences could be ascribed to the inherent characteristics of the genome of the strain used in each experimental model. It is known that the adaptation of the BA71V strain to replicate in Vero cells includes the deletion of large genomic areas in both the left and right variable regions of the virus genome, producing the deletion of 11 genes belonging to the MGF360/505 gene families [36]. Those severe changes in the genome could produce the elimination of genes that may be implicated in a partial or complete replacement of the function of the gene under study. Conversely, the ASFV-G strain used here as the parental virus is a field isolate without significant genomic alteration. Therefore, the function of the *O174L* gene may be supplied by any other virus gene hypothetically having a vicariant function.

The potential importance of O174L in virus replication has led to studies on potential targeting for biotherapeutics to inhibit virus replication [21]. The results presented here suggest that targeting O174L expression as a therapeutic approach to prevent or abort ASFV infection in pigs will not be successful.

In summary, we determined that O174L is a non-essential protein since its deletion from the ASFV-G genome does not significantly alter virus replication in swine macrophage cultures, and its deletion from the ASFV-G isolate causes no decrease in virus virulence in domestic pigs.

The identification of ASFV genes involved in virulence has been shown as essential in the development of attenuated virus strains. Therefore, their correct characterization and genetic manipulation is the first step in designing and producing recombinant ASF, live attenuated vaccine candidates. The introduction of ASFV strains with an altered O174L protein into Germany led to the detection of a surprisingly high genetic diversity among strains of different geographical areas [37]. It remains to be evaluated whether or not O174L plays a role in mutation rate and thus viral evolution.

## Data Availability

All data are included in the manuscript.
